# Nutritional Value of Female *Eriocheir sinensis* from Three Different Habitats in the Lower Reach of the Yangtze River with a Special Emphasis on Lipid Quality

**DOI:** 10.3390/foods14142434

**Published:** 2025-07-10

**Authors:** Lizhi Yu, Xueqian Guo, Mingyu Yin, Xichang Wang

**Affiliations:** 1 College of Food Science and Technology, Shanghai Ocean University, Shanghai 201306, China; yulizhi2012@126.com; 2 College of Tea and Food Science, Wuyi University, Wuyishan 354300, China; 3 Department of Food Science &Technology, School of Agriculture and Biology, Shanghai Jiao Tong University, Shanghai 200240, China; guoxueqian@sjtu.edu.cn

**Keywords:** *Eriocheir sinensis*, habitats, lipid class composition, fatty acid, lipid quality indices

## Abstract

The cultured habitat of *Eriocheir sinensis* is a crucial factor influencing its nutritional quality. Therefore, it is essential to clarify the differences in the nutritional quality of *Eriocheir sinensis* reared in different habitats. This study investigated and compared the nutritional value of three edible parts (the hepatopancreas, gonads, and muscles) of female *Eriocheir sinensis* from three different habitats in the lower reach of the Yangtze River, with a special emphasis on lipid compounds. In addition to tissue indices, proximate composition, energy content, lipid classes, and fatty acid profile, eight lipid quality indices were proposed to evaluate the lipid nutritional quality. The results indicated that the *Eriocheir sinensis* from the three different habitats were all in good developmental condition. No significant differences were observed for the hepatopancreas index (HIS), gonadosomatic index (GSI), and total edible yield (TEY) among the three habitats, except for muscle index (MI), which was significantly higher in the L-crabs and E-crabs compared to the P-crabs. The highest protein content was found in the gonads, while the hepatopancreas had the highest crude lipid content. Regarding lipid classes, triglycerides dominated the hepatopancreas, and phospholipids were predominant in muscles, whereas phospholipids and triglycerides were predominant in approximately equal amounts in the gonads. Taking eight lipid quality indices into account together, the three major edible tissues of *Eriocheir sinensis* from the estuarine habitat had the highest nutritional value, followed by the hepatopancreas from the pond habitat. The current research will provide basic nutritional data for consumers to purchase *Eriocheir sinensis* and establish the theoretical groundwork for paving new paths for improving the nutritional quality combined with habitat conditions in future studies.

## 1. Introduction

*Eriocheir sinensis* is a small crab species, commonly called the Chinese mitten crab, and it is native to coastal and estuarine habitats from the Fujian province in China (26°N) to the Korean Peninsula (40°N) [1,2]. Although the Chinese mitten crab is considered an invasive species in the European and American regions [3,4], it is one of the most important economic aquatic animals worldwide, especially in East Asia [5]. In China, *Eriocheir sinensis* is a traditional aquatic species and widely distributed, with production reaching about 888,629 tons in 2023 [6], especially in the Yangtze River Basin [7]. In recent years, with the continuous improvement of breeding technology and aquacultural practices, the farming of *Eriocheir sinensis* has spread across China [8]. However, the lower reaches of the Yangtze River are still the main farming areas for *Eriocheir sinensis* in terms of their farming sizes and economic benefits [9,10]. *Eriocheir sinensis* is the most beloved and consumed crab because of its rich nutritional profile, palatable taste, and its unique and pleasant aroma [11].

The hepatopancreas, gonads, and muscles were the three main edible biological tissues of *Eriocheir sinensis* [12]. Studies on human dietary habits have demonstrated that the consumption of aquatic products is beneficial to health, mainly because aquatic products provide almost all the fatty acids that are beneficial to human health, especially n-3 PUFA [13]. The n-3 family of long-chain polyunsaturated fatty acids (n-3 LC-PUFAs) is a key nutrient whose beneficial effects on human health are well known [14]. Due to their physiological and nutritional roles in all life stages, eicosapentaenoic acid (EPA, 20:5n-3) and docosahexaenoic acid (DHA, 22:6n-3) are the most recognized n-3 LC-PUFAs, which cannot be synthesized by humans and must be obtained from the diet [15]. Lipids are nutritionally significant in crustaceans; a number of studies have investigated the functional properties of lipid fractions and fatty acid profiles, but the implications of lipid quality indices for human health have been scarcely investigated. Moreover, some lipid quality indices are also important indicators for evaluating the fatty acid profile [16,17]. This study proposed using different lipid quality indices to evaluate the nutritional value of *Eriocheir sinensis* based on their fatty acid profile.

The nutritional quality of *Eriocheir sinensis* was usually evaluated through the content and compositional analyses of conventional nutrients in the aforementioned three edible tissues. The nutritional value of *Eriocheir sinensis* varied with different edible tissues and sourced habitats [18]. Lakes and ponds are the traditional farming habitats for *Eriocheir sinensis*. The Chinese mitten crab from the Yangcheng Lake has been the most renowned for a long time [19]. In the Yangtze River estuary, which is situated at the intersection of the Yangtze River and the East China Sea, there is a slightly higher salinity compared to freshwater areas. The Chinese mitten crab is a euryhaline crustacean, and it has been demonstrated that salinity can affect the lipid composition and flavor quality of the Chinese mitten crab [20,21]. The nutritional quality of lake-stocked *Eriocheir sinensis* was found to be superior to that of pond-reared ones based on a comparison of their morphological traits, tissue indices, and the proximate and fatty acid compositions of their gonads, muscles, and hepatopancreas [22]. It was speculated that the low biodiversity of microalgae and flourishing macrophytes in the lakes contributed directly or indirectly to the quality difference of the Chinese mitten crab compared to in the ponds [23]. Additional studies have found that the quality of *Eriocheir sinensis* from lakes and rivers varied due to different natural ecosystems, such as microorganisms, aquatic plants, etc. [24,25]. Although there have been many studies on the quality of *Eriocheir sinensis* from different habitats, limited studies have been conducted to assess the nutritional value of *Eriocheir sinensis* using the lipid quality indices.

The nutritional quality of *Eriocheir sinensis* is significantly influenced by its cultural habitat conditions. Therefore, considering the differences in the three habitats, namely, lakes, estuaries, and ponds in the lower reach of the Yangtze River, the objective of this study was to compare and assess the differences in tissue indices and proximate composition, especially for the nutritional value of the fatty acid profile of three edible tissues of *Eriocheir sinensis* from three different habitats. The results of this study will provide nutritional reference information for consumers to make informed choices when purchasing *Eriocheir sinensis*. Meanwhile, based on the present farming parameters, the results will also lay a theoretical and technical foundation for improving the nutritional value of *Eriocheir sinensis* by modifying habitat conditions in future studies.

## 2. Materials and Methods

### 2.1. Study Areas and Sampling Methods

**Figure 1:**
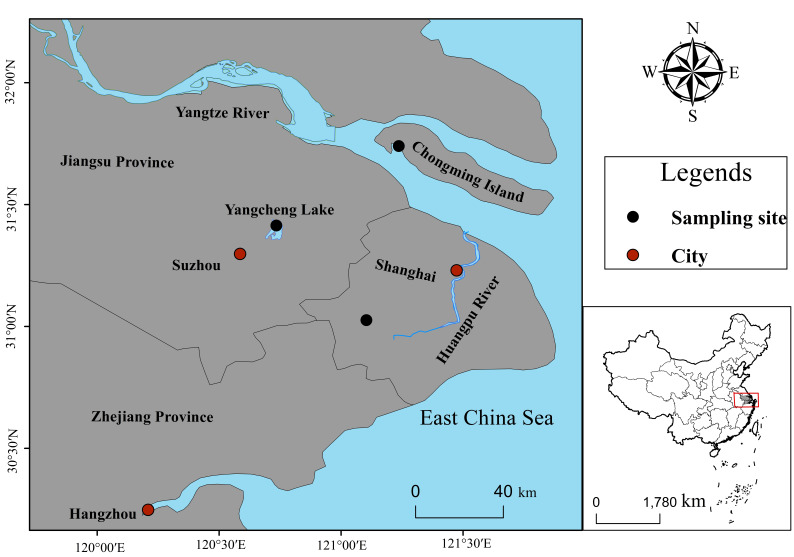
Location of the sample sites of *Eriocheir sinensis* (black points).

The lower reaches of the Yangtze River, located in East China and bordering the East China Sea, are one of the main breeding areas for the Chinese mitten crab. Specifically, the three different habitat sources are located in the Yangcheng Lake, the Yangtze River estuary of Chongming Island, and the upper reaches of the Huangpu River. Correspondingly, the crab samples were reared in the lake, the Yangtze River estuary, and pond waters, respectively; accordingly, these samples were named L-crabs, E-crabs, and P-crabs, respectively. The geographic locations of the collected *Eriocheir sinensis* are shown in Figure 1. Except for the different habitats, all experimental crabs belonged to the same genetic species and were raised using the same cultured practices.

In November 2023, 90 female samples were collected from 3 sampling sites, each containing 30 individuals. Each crab was of a commercially appropriate size (150 ± 10 g). The practice of the sampling plans for the crabs was conducted according to the Chinese Standard GB/T 30891-2014 [26]. At every sampling site, 30 live female samples were collected randomly and transported to the Laboratory of Food Nutrition and Quality Evaluation, Shanghai Ocean University, within two hours. To avoid cannibalism, each crab was tied separately with a cotton rope and placed in a perforated plastic box (20 cm × 30 cm × 40 cm) with ice bags to keep the lower temperature.

### 2.2. Sample Preparation and Tissue Indices

All crabs were kept in a refrigeration house (5–8 °C) to decrease their metabolism and then washed carefully through water rinsing and stunned before being euthanized by piercing their two nerve centers using a stainless steel rod. As advised by the Codex Alimentarius Commission, the rod was placed through the vent and one of the eyes [27]. Next, the crabs were dissected to obtain the hepatopancreas and ovaries, and the meat from the whole body was carefully removed and collected. The gonads, hepatopancreas, and meat of each crab were then weighed separately. Finally, all collected samples were stored at −40 °C for later biochemical analyses. The tissue indices were calculated using equations, as shown in Table 1, respectively.

**Table 1:** List of tissue indices and their mathematical expressions.

Tissues Indices	Mathematical Expression	No.
Hepatopancreas index	$HSI\left(\%\right)=\frac{hepatopancreas\;wet\;weight}{body\;wet\;weight}\times 100$	(1)
Gonadosomatic index	$GSI\left(\%\right)=\frac{gonad\;wet\;weight}{gonad\;wet\;weight}\times 100$	(2)
Muscle index	$MI\left(\%\right)=\frac{meat\;wet\;weight}{body\;wet\;weight)}\times 100$	(3)
Total edible yield	$TEY\left(\%\right)=MY+HSI+GSI$	(4)
Condition factor	$CF(g/{cm}^{3})=\frac{body\;wet\;weight}{{carapace\;length}^{3}}\times 100$	(5)

### 2.3. Proximate Composition Determination and Energy Content

Before the biochemical analysis, the three edible tissues from each habitat were pooled and homogenized separately. The moisture, total ash, crude protein, and total lipid contents were determined according to the AOAC methods [28], while the total carbohydrate content in the samples was measured using the phenol-sulfuric acid method, as described in detail by [29]. The results were expressed in % of wet weight. The energy content was estimated as follows: proteins, 4.27 kcal g^−1^ of wet weight; lipids, 9.02 kcal g^−1^ of wet weight; and carbohydrates, 4.11 kcal g^−1^ of wet weight (1 kcal = 4184 kJ) [30].

### 2.4. Lipid Classes and Fatty Acid Profile Analysis

Firstly, a chloroform–methanol solution (2:1, *v*/*v*) was used to extract the total lipids [31]. The lipid classes were analyzed based on previous methods [32]. Briefly, the separation of the lipid fractions was carried out using a hexane/diethyl ether/formic acid (42/28/0.3, *v*/*v*/*v*) solvent system. The resulting lipid classes were quantified for total phospholipids (PLs), triacylglycerols (TAGs), free fatty acids (FFAs), and cholesterol (CHO) using an Iatroscan MK-6 s TLC-FID analyzer (Iatron Laboratories Inc., Tokyo, Japan). The level of each lipid class was expressed as a percentage of total lipid classes (%).

The fatty acid methyl esters (FAMEs) were prepared using a slightly modified method [33]. In short, 5 mL of methanolic NaOH (0.5 mol/mL) was mixed with 100 μL of internal standard (C19:0, 10 mg/mL) and 0.1 g of the total lipid samples. Then, the mixture was heated to 100 °C and held for 10 min on a condensing and concentrating apparatus (HWS24, HongLang, Zhengzhou, Henan, P.R. China). Following that, the mixture was stirred for 3 min at 100 °C with 3 mL of boron trifluoride–methanol (14% in methanol) added, and then it was held at 100 °C for 2 min with the addition of 2 mL of n-hexane. Lastly, 10 mL of saturated NaCl solution was added to the mixture. After cooling the mixture to room temperature (24–27 °C), the upper n-hexane layer was collected using a 2 mL disposable syringe, placed in a 2 mL thread screw neck vial with a septum (32 × 11.6 mm, ANPEL Inc.), and filtered using a nylon syringe filter (13 mm × 0.22 µm) for further analyses.

The fatty acid composition of the total lipids was determined by a GC analysis. A gas chromatograph TRACE GC ULTRA (Thermo Fisher Inc., Waltham, MA, USA) fitted with a flame ionization detector (Thermo Fisher Inc.) and an Agilent (Santa Clara, CA, USA) SP-2560 capillary column (100 m length × 250 µm internal diameter, 0.2 µm of film) was used. The temperatures that the chromatographic columns were programmed to reach were as follows: the initial temperature was 70 °C, then heated to 140 °C (20 °C/min) and held for 1 min; to 180 °C (4 °C/min) and held for 1 min; and to 225 °C (3 °C/min) and held for 30 min. The gasification temperature was 250 °C. The flow rate of N_2_ was 1 mL/min. The injection volume was 1 μL, with a split ratio of 45:1. FAME were identified by comparison of their retention time with the standard mixture. The contents of different fatty acids were determined using the area ratio of the GC peak between internal standard C19:0 and the different fatty acids being tested. The specific calculation formula is as follows:

(6) $$X_{i}=F_{i}\times \frac{A_{i}}{A_{C_{19:0}}}\times \frac{C_{C_{19:0}}\times V_{C_{19:0}}\times 1.047}{m}\times 100\times F_{FAMEi-FAi}$$

where *X_i_* is the content of different fatty acids, mg/100 g; *F_i_* is the response factor of each FAME; *A_i_* is the peak area of each FAME in the sample; *A_C19:0_* is the peak area of the internal standard C19:0; *C_C19:0_* is the concentration of C19:0, mg/mL; *V_C19:0_* is the volume of the internal standard C19:0, mL; 1.047 is the transfer coefficient of C19:0 to C19:0 FAME; *F_FAMEi−FAi_* is the transfer coefficient of FAME for each fatty acid; and *m* is the mass of total lipids, g.

(7) $$F_{i}=\frac{C_{si}\times A_{19:0}}{A_{si}\times C_{19:0}}$$

where *C_Si_* is the concentration of each FAME in the mixed standard, mg/g; *A_19:0_* is the peak area of the C19:0 FAME standard; *A_Si_* is the peak area of each FAME in the mixed standard; and *C_19:0_* is the concentration of the C19:0 FAME standard, mg/g.

Fatty acid composition was expressed as mg/g of the total lipids.

### 2.5. Lipid Quality Indices

Eight nutritional quality indices were calculated in three edible tissues of *Eriocheir sinensis* by means of the following formulae provided in Table 2.

**Table 2:** List of lipid quality indices and their mathematical expressions.

Quality Index	Mathematical Expression	No.
Polyunsaturated to saturated fatty acid ratio	PUFA/SFA = (∑PUFA)/(∑SFA)	(8)
Omega-3/omega-6 ratio	n-3/n-6 = $\frac{\sum (n-3)PUFA}{\sum (n-6)PUFA}$	(9)
Fish lipid quality	$FLQ=100\times (\frac{22:6+20:5}{\Sigma FA})$	(10)
Atherogenicity index	$AI=\frac{\left[12:0+(4\times 14:0)+16:0\right]}{\sum MUFA+\sum PUFA(n-6)+(n-3)}$	(11)
Thrombogenicity index	$TI=\frac{\left[14:0+16:0+18:0\right]}{\left[(0.5\times \sum MUFA)+(0.5\times \sum PUFA(n-6)+(3\times \sum PUFA(n-3)+(\frac{\sum n-3}{\sum n-6})\right]}$	(12)
Hypo- to hypercholesterolemic ratio	H H = ( c i s − 18 : 1 + Σ P U F A ) ( 12 : 0 + 14 : 0 + 16 : 0 )	(13)
Health-promoting index	H P I = ∑ U F A [ 12 : 0 + 14 : 0 × 4 + 16 : 0 ]	(14)
Nutritive value index	N V I = 18 : 0 + 18 : 1 16 : 0	(15)

PUFA/SFA, n-3/n-6, FLQ, AI, TI, HH, and HPI were calculated according to [24]. NVI was calculated according to [34].

### 2.6. Statistical Analysis

All samples and assays were carried out thrice (*n* = 3), except for biological tissue indices, which were duplicated tenfold (n = 10). The values were presented as average ± standard deviation. All differences among samples were considered to be statistically significant at *p* < 0.05 using a one-way analysis of variance (ANOVA), followed by a Tukey’s post hoc test using SPSS 20.0 (SPSS Chicago, IL, USA). A principal component analysis (PCA) biplot and a hierarchical cluster analysis (HCA) were accomplished by XLSTAT 2019 (Addinsoft Inc., New York, NY, USA), and the PCA standardization used during the computations was based on Pearson’s correlation. Origin 2025 (Origin Lab Corporation, Northampton, MA, USA) was used to create and process the images.

## 3. Results and Discussion

### 3.1. Tissue Indices

**Figure 2:**
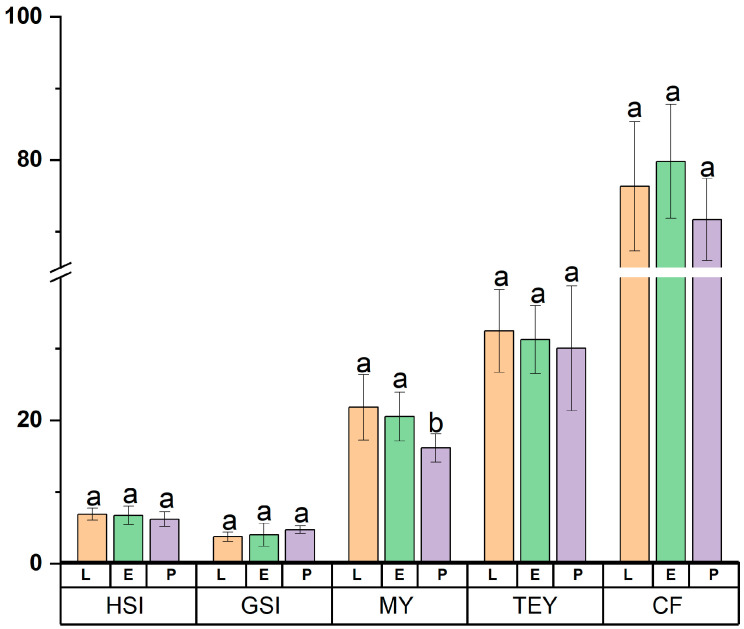
Tissue indices of *Eriocheir sinensis* from three different habitats. The values of certain tissue indices that do not share the same lowercase are statistically significantly different (*p* < 0.05); L, lake; E, estuary; P, pond.

The determination of the tissue indices in crustaceans, such as *Eriocheir sinensis*, is of biological and technological interest [35]. The present study highlights tissue index differences for *Eriocheir sinensis* from three typical habitats. As is illustrated in Figure 2, there were no significant differences in the HIS and GSI indices of female crabs from the three habitats, and MI was significantly higher in L-crabs and E-crabs than in P-crabs, but there were no significant differences in TEY from the three different habitat sources. Fulton’s condition factor is widely used in fisheries and general fish biology studies as an indicator of the “condition”, “well-being”, “plumpness”, etc. [36]. Herein, in terms of CF, there were also no significant differences among the three habitat sources, indicating that the *Eriocheir sinensis* sampled in this study were homogeneous and in good growth condition. In addition, as can also be seen in Figure 1, among the three edible tissues (HSI, GSI, and MI), they all conformed to the following order: MI > HIS > GSI, regardless of their habitat sources.

The nuances in these parameters may be attributed to the different cultured conditions of the three habitats. Currently, the classification of different grades of commercial *Eriocheir sinensis* and the factors consumers consider when purchasing crabs are mainly based on the body weight of *Eriocheir sinensis* and their corresponding tissue indices [37]. Therefore, it is of great importance that tissue indices are available to consumers and farmers for making comparisons related to the habitats, as they may provide basic reference data.

### 3.2. Proximate Composition and Energy Content

Table 3 shows the proximate composition and energy content of female *Eriocheir sinensis* from three different habitats in the lower reach of the Yangtze River. There were no significant differences in the moisture, total lipids, total carbohydrates, and energy values in the edible hepatopancreatic tissues from the three habitat sources. However, the total ash content of P-crabs was significantly lower than that of L-crabs and E-crabs. The opposite was true for crude protein in P-crabs, which was significantly higher than the other two. In gonadal tissues, there were no significant differences in total ash and crude protein among the three habitat sources of the crabs. Total lipids and total carbohydrates had the same characteristics, with P-crabs having the lowest levels, significantly lower than E-crabs and L-crabs. In crab meat, moisture or crude protein content among the three habitats of *Eriocheir sinensis* did not differ significantly. The total lipid and energy values followed the same pattern, with P-crabs having the highest values and significantly higher values than E-crabs and L-crabs.

Regardless of the habitat of *Eriocheir sinensis*, from the perspective of the three major edible tissues, the hepatopancreas had the highest total lipid content, the gonads had the highest protein content, and the muscle had the highest total carbohydrate content. This is in line with previous findings [18,21]. These data provided a basis for choosing different edible tissues according to our nutritional needs when eating crabs.

### 3.3. Lipid Classes

**Figure 3:**
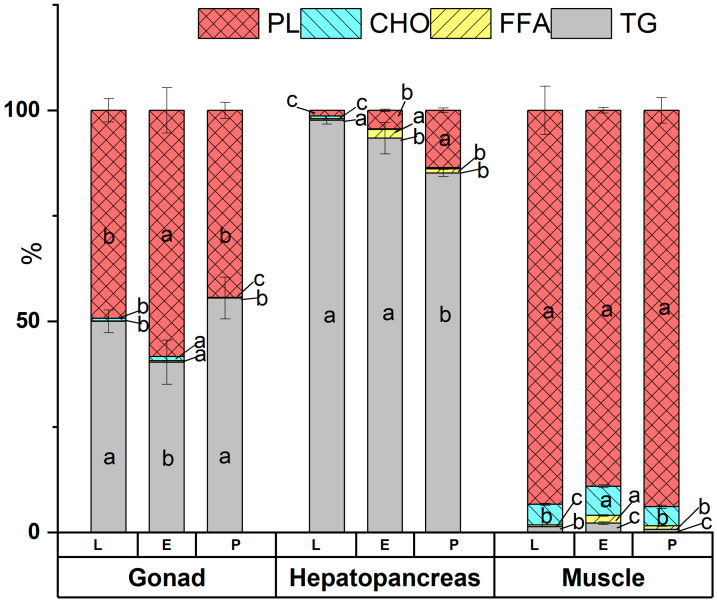
Main lipid classes of different edible tissues in female *Eriocheir sinensis* (%total lipids). A certain lipid class of the same edible tissues sharing different lowercases are significantly different (*p* < 0.05). L, lake; E, estuary; P, pond.

Four lipid classes, namely, TG, FFA, CH, and PL, were detected by TLC-FID in the three edible tissues, and the results are presented in Figure 3. In the gonads, PL and TAG represented the main lipid classes, while they were low in both FFA and CHO, with <1% of the total lipids. The levels of TG content were noticeably greater in the P- and L-crabs compared to the E-crabs (*p* < 0.05). On the contrary, the highest amount of PL was found in E-crabs, which was significantly higher than in L-crabs and P-crabs (*p* < 0.05). Lipids in the hepatopancreas tissues were mainly in the form of TGs (>85%), followed by PL. FFAs and CHOs were the least abundant (<1%). The lowest TG content was found in P-crabs, which was significantly lower than in L-crabs and E-crabs (*p* < 0.05). When it comes to PL content, there were noticeable variations in the hepatopancreas tissues from the three different sources. The pond crabs had higher levels of PL content compared to the estuarine and lake crabs. When it comes to PL content, the contents of the three in descending order are as follows: P-crabs, E-crabs, and L-crabs (*p* < 0.05). In crab meat, the lipids were mainly in the form of PLs (>90%), followed by CHO (approximately 5%). TG and FFA were both low. There were no significant differences found in the PL contents of crab meat from the three habitats. Crabs living in estuaries had a noticeably greater amount of CHO compared to the other two habitats. Regardless of the source, the different edible tissues exhibited different lipid class profiles, which was in agreement with previous studies [32].

### 3.4. Fatty Acid Profile

The fatty acid content in the three edible tissues of *Eriocheir sinensis* from the three different habitats is listed in Table S1 (see Supplementary Table S1 for detailed data). In this experiment, 33 fatty acid molecules were detected in total. Unsaturated fatty acids (UFAs) were dominant in all three tissues of the three habitat-sourced crabs. However, the fatty acid composition varied depending on the different edible tissues and sourced habitats of E. sinensis, which reflected the effect of sourced habitats on the quality of *Eriocheir sinensis*.

In the hepatopancreas, the most abundant saturated fatty acid (SFA) was found in L-crabs, followed by P-crabs and E-crabs (*p* < 0.05). P-crabs and L-crabs have higher MUFA content compared to E-crabs (*p* < 0.05). Higher PUFA contents were found in L-crabs compared to E-crabs and P-crabs. The intake of PUFA is important for human health. However, n-3 and n-6 PUFA fatty acids must be obtained dietarily through food because humans lack the specific enzymes to create double bonds at these positions [38]. Of the PUFAs, the two most important fatty acids in all tissues analyzed were EPA and DHA. There is evidence that DHA and EPA can help lower the risk of cancer and coronary heart disease [39]. The daily intake of EPA and DHA is 250 mg day^−1^ for adults, which is recommended by FAO/WHO [40]. Similar to MUFAs, P-crabs and L-crabs have higher EPA+DHA content compared to E-crabs (*p* < 0.05). In the gonads, the SFA content of the three habitat-sourced Eriocheir sinensis was the same as in the hepatopancreas. As for MUFAs, the highest level was found in P-crabs, followed by E-crabs and L-crabs (*p* < 0.05). However, P-crabs’ gonads possessed the highest content of PUFAs and EPA+DHA. In the meat of E. sinensis from all three habitats, P-crabs and L-crabs have higher SFA, PUFA, and EPA+DHA content compared to E-crabs (*p* < 0.05). However, higher MUFA content was found in E-crabs compared to the other two (*p* < 0.05). In comparison to the hepatopancreas and gonads, the abdomen was low in lipid and high in protein content tissues, and another study also found the same result [41].

**Figure 4:**
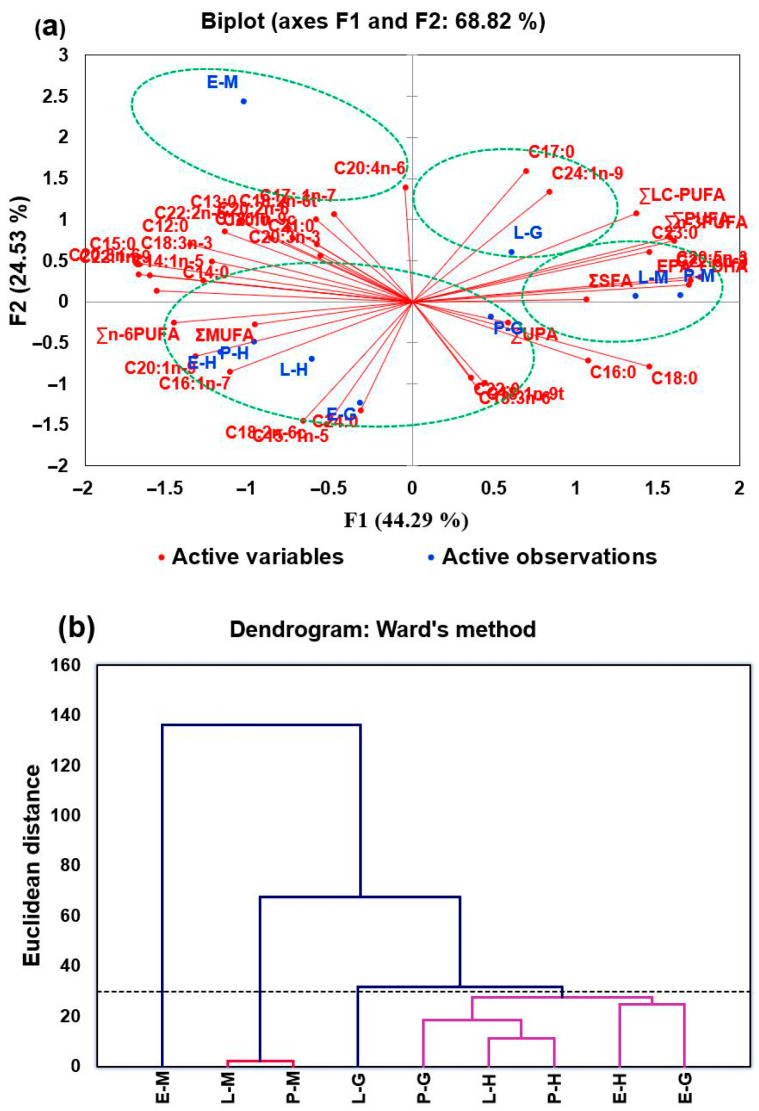
PCA biplot (**a**) and HCA (**b**) based on the fatty acid profile of different edible tissues of female *Eriocheir sinensis* from three different habitats. P-H, L-H, and E-H: hepatopancreas from pond, lake, and estuary habitats, respectively. P-G, L-G, and E-G: gonads from pond, lake, and estuary habitats, respectively. P-M, L-M, and E-M: muscles from pond, lake, and estuary habitats, respectively.

PCA and HCA were used to further compare the differences and similarities in fatty acid composition among the different edible parts. The PCA biplot for the fatty acid composition of the different edible tissues (Figure 4a), in which each red point represents a fatty acid variable and each blue point denotes an edible tissue, clearly evidences the differences among the different edible tissues and the relationship between edible tissues and fatty acids. The first and second principal components accounted for 68.82% of the variation (44.29% and 24.53%, respectively) (Figure 4a). In the PCA biplot, the angle between the sample point and the fatty acid point represents the magnitude of the corresponding fatty acid content value in the sample; the smaller the angle, the greater the corresponding fatty acid content value; for example, C22:6n-3 was found to be higher in E-M samples (see Table S1). Meanwhile, the closer the distance between two different samples, the more similar their fatty acid profiles are, and they may be grouped together as one cluster. Nine samples could be clearly classified into four groups based on fatty acid composition (Figure 4a), which was in line with the HCA results (Figure 4b). Only E-M was located in the second quadrant, far from the other edible tissues (Figure 4a), and is, therefore, clustered in a separate category (Figure 4b), and this is also the case for L-G, which was located in the upper part of the first quadrant. E-H, P-H, L-H, and E-G were located in the third quadrant (Figure 4a), and they were clustered into one category (Figure 4b). The other three edible tissues were clustered together. It was concluded that the fatty acid composition of the hepatopancreas of female *Eriocheir sinensis* from different habitats had less variation, which was exactly the opposite for the gonads, in which there was a large variation in fatty acid composition. The muscles of *Eriocheir sinensis* from the estuary differed greatly from lake and pond habitats, which had similar fatty acid profiles.

### 3.5. Lipid Quality Indices

The nutritional value of dietary food is generally assessed using nutritional indices [42]. This study proposed using different lipid quality indices to evaluate the nutritional value of *Eriocheir sinensis* based on their fatty acid profile. Eight lipid quality indices were presented in Table 4. These calculated fatty acid ratios characterize the quality of lipids in tissues from different perspectives. The excessive intake of SFA has been reported to be undesirable because it is associated with elevated serum levels of total cholesterol and LDL cholesterol. Meanwhile, dietary PUFAs, e.g., EPA and DHA, can also reduce cholesterol absorption. In order to keep a healthy cardiovascular status, a PUFA/SFA ratio above 0.40 is desirable [43]. Aquatic products are considered an excellent source of healthy lipids because of their higher n-3 polyunsaturated fatty acid (PUFA) content compared to other animal food sources. Since high levels of n-6 PUFA may promote inflammatory diseases, normal diets typically contain lower amounts of n-3 PUFA than n-6 PUFA [44]. FAO/WHO recommended that the appropriate ratio of n-3/n-6 PUFA be 0.1–0.2 [45]. If the ratio is greater than 0.2, it is more beneficial to human health [46]. Diets rich in n-3 polyunsaturated fatty acids, mainly EPA and DHA, are thought to be beneficial for the prevention and treatment of a variety of diseases, including cardiovascular disease and inflammation [47]. The FLQ calculates the sum of EPA and DHA as a percentage of total fatty acids, which was originally used to assess the quality of the fish lipids and is more suitable for marine products because of their high proportions of EPA and DHA [48]. Previous studies have reported the quality of fish fat through the FLQ index, with values ranging from 13.01 to 36.37 [49]. In the present study, the PUFA/SFA contents were all greater than 0.4, with the highest ratio, especially in crab meat. The ratios of n-3/n-6 were also all greater than 0.2, and, similarly, the proportion of body meat was the largest. However, the ratios obtained for the swimming crab Portunus trituberculatus [50] are all greater than those obtained in this study. Hepatopancreatic tissue has the lowest FLQ value, and, in comparison, the gonads and flesh of lake and pond crabs have higher FLQ values. By combining these three ratios described above, the lipid nutrient quality was highest in the gonads and crab meat, particularly from the lake and pond habitats.

AI characterizes the atherogenic potential of FAs, indicating the relationship between the sum of SFAs and the sum of UFAs, whereas TI characterizes the thrombogenic potential of FAs, indicating the tendency to form clots in blood vessels and contributing to different FAs. The ratio HH is directly related to cholesterol metabolism and predicts the cardiovascular risk and characterizes the relationship between hypocholesterolemic fatty acid (C18: 1 and PUFA) and hypercholesterolemic FA, first proposed by Santos-Silva et al. in 2002 [51]. The HPI, the inverse of the AI, was proposed to assess the nutritional value of dietary fat [52], which focuses on the effect of FA composition on cardiovascular diseases (CVDs). Previous research suggested that only C16:0 increases blood cholesterol, whereas C18:0 has no effect, and C18:1 decreases blood cholesterol content. Because these fatty acids represent the majority of fatty acids, the ratio of (C18:0 + C18:1)/C16:0 could perhaps better describe the possible health effects of different types of lipids [53]. Therefore, the NVI value mainly describes the effect of fatty acids on blood cholesterol levels. A healthy diet was characterized by low AI and TI indices, as well as high HH, HPI, and NVI indices [47]. In the hepatopancreas, the AI and TI indices were significantly higher in the L-crabs, while there were no significant differences between the E- and P-crabs. HH and HPI followed the following trend: E-crabs > P-crabs > L-crabs (*p* < 0.05). In the case of NVI, E-crabs and P-crabs presented with higher values compared to L-crabs. Combining the above five indicators, E-crab and P-crab hepatopancreas were found to be higher in lipid nutrient quality.

Concerning the gonads, the AI and TI of the L-crabs were significantly higher compared to the E-crabs and P-crabs, with no significant difference between the latter two. The P-crabs had significantly lower HH values compared to the L-crabs and E-crabs, but no significant difference was observed between the latter two. The E-crabs and P-crabs have a significantly higher HPI value compared to the L-crabs, but no significant difference was observed between the former two. The NVI values were not statistically significantly different among the three sources of crabs (*p* > 0.05). In conclusion, the gonads from the estuarine habitat presented the highest lipid nutritional value. In the muscle tissues, the E-crabs had a significantly lower AI and AI as well as higher HH, HPI, and NVI indices compared to the P-crabs and L-crabs, with no significant differences between the latter two. Therefore, crab meat from the estuary habitat possessed the highest lipid nutritional value.

Overall, in terms of lipid nutrient quality, the results of the present study indicated that the three major edible tissues of crabs from the estuarine habitat had the highest nutrient value, which may be related to the fact that estuarine waters have relatively high salinity, resulting in the three major edible tissues containing high levels of UFA, especially PUFA [54]. Additionally, P-crabs’ hepatopancreas was also nutritious in lipid nutrient quality. Five lipid quality indices, n-3/n-6, AI, TI, PUFA/SFA, and FLQ, were used to assess the nutritional value of invasive Eriocheir sinensis from the Odra Estuary (Baltic Basin) [1]. Compared to the Yangtze River estuary, higher AI, PUFA/SFA, and FLQ and lower TI in the hepatopancreas, higher n-3/n-6, PUFA/SFA, and FLQ and lower AI and TI in the gonads, higher AI, TI, PUFA/SFA, and FLQ and lower n-3/n-6 in the muscles were observed in the female *Eriocheir sinensis*. Further research is required to evaluate the nutritional value of *Eriocheir sinensis* using the lipid quality index.

It should be noted that this study only focused on female *Eriocheir sinensis* from three different habitats. Under the influence of seasonal variation, gender differences, and other factors, the nutritional quality of Chinese hairy crabs also needs to be studied. In addition, future research must continue to be conducted under precisely controlled environmental conditions in laboratories.

## 4. Conclusions

This study elucidated and compared the nutritional value of female *Eriocheir sinensis* from three different habitats in the lower reach of the Yangtze River with a special emphasis on lipid quality, including lipid classes, fatty acid profile, and lipid quality indices. As for the lipid classes, triglycerides were dominant in the hepatopancreas, the muscles were predominated by phospholipids, and lipids in the gonads were dominated by triglycerides and phospholipids in approximately equal amounts. The fatty acid profile indicated that *Eriocheir sinensis* possessed a high nutritional value, considering the important benefits of n-3 PUFAs for human health. Eight nutritional quality indices were calculated, and the results indicated that the three major edible tissues of crabs from the estuarine habitat had the highest nutrient value, followed by P-crabs’ hepatopancreas in terms of lipid nutritional quality. The present study provided nutritional data on female Chinese mitten crabs reared in different habitats. Future research will focus on the relationship between the environmental parameters of different habitats and the nutritional quality indices of cultured *Eriocheir sinensis* and explore new pathways to improve the nutritional quality of *Eriocheir sinensis* by modifying the habitat conditions.

## Figures and Tables

**Table 3:** Proximate composition and energy content in the hepatopancreas, gonads, and muscle for three different habitat female *Eriocheir sinensis* in the lower reach of the Yangtze River.

Indices	Lake	Estuary	Pond
Hepatopancreas			
Moisture (%)	53.18 ± 0.57 ^a^	46.42 ± 3.20 ^a^	53.12 ± 8.92 ^a^
Total ash (%)	1.33 ± 0.09 ^a^	1.18 ± 0.14 ^ab^	1.08 ± 0.05 ^b^
Total lipid (%)	34.02 ± 2.37 ^a^	35.42 ± 2.11 ^a^	33.65 ± 4.84 ^a^
Crude protein (%)	7.68 ± 0.45 ^b^	6.89 ± 0.78 ^b^	9.24 ± 1.19 ^a^
Total carbohydrate (%)	0.73 ± 0.33 ^a^	0.46 ± 0.01 ^a^	0.47 ± 0.04 ^a^
Energy (Kcal per 100 g)	342.64 ± 21.62 ^a^	350.82 ± 19.42 ^a^	344.87 ± 40.03 ^a^
Gonads			
Moisture (%)	53.55 ± 1.09 ^a^	45.16 ± 3.42 ^b^	49.15 ± 1.96 ^ab^
Total ash (%)	2.46 ± 0.36 ^a^	2.26 ± 0.07 ^a^	2.14 ± 0.07 ^a^
Total lipid (%)	7.34 ± 0.81 ^a^	6.90 ± 0.36 ^a^	4.94 ± 0.22 ^b^
Crude protein (%)	28.83 ± 1.63 ^a^	27.12 ± 1.59 ^a^	29.02 ± 1.39 ^a^
Total carbohydrate	1.39 ± 0.26 ^a^	1.35 ± 0.08 ^a^	0.14 ± 0.03 ^b^
Energy (Kcal per 100 g)	195.08 ± 13.08 ^a^	183.63 ± 3.51 ^ab^	169.05 ± 7.76 ^b^
Muscles			
Moisture (%)	75.74 ± 2.30 ^a^	74.41 ± 2.24 ^a^	74.75 ± 2.42 ^a^
Total ash (%)	2.17 ± 0.06 ^a^	1.88 ± 0.11 ^b^	2.04 ± 0.03 ^a^
Total lipid (%)	0.20 ± 0.07 ^b^	0.59 ± 0.18 ^b^	0.79 ± 0.39 ^a^
Crude protein (%)	18.05 ± 1.59 ^a^	16.98 ± 1.04 ^a^	17.44 ± 1.39 ^a^
Total carbohydrate(%)	1.11 ± 0.06 ^a^	0.78 ± 0.20 ^b^	0.33 ± 0.03 ^c^
Energy (Kcal per 100 g)	83.48 ± 6.38 ^b^	81.09 ± 5.73 ^b^	109.07 ± 8.14 ^a^

Values in the same line that do not share the same lowercase are statistically significantly different (*p* < 0.05).

**Table 4:** Values of the lipid quality indexes in the hepatopancreas, gonads, and muscles for three different habitats of female *Eriocheir sinensis* in the lower reaches of the Yangtze River.

Quality Indices	Hepatopancreas			Gonads			Muscles		
	Lake	Estuary	Pond	Lake	Estuary	Pond	Lake	Estuary	Pond
PUFA/SFA	1.45 ± 0.05^a^	0.86 ± 0.04 ^c^	1.26 ± 0.10 ^b^	0.59 ± 0.02 ^b^	1.25 ± 0.04 ^a^	1.25 ± 0.02 ^a^	1.70 ± 0.07 ^b^	1.84 ± 0.05 ^a^	1.66 ± 0.05 ^b^
n-3/n-6	1.02 ± 0.03 ^a^	0.61 ± 0.03 ^b^	1.28 ± 0.37 ^a^	2.44 ± 0.14 ^a^	1.18 ± 0.12 ^c^	2.16 ± 0.11 ^b^	4.07 ± 0.08 ^b^	1.88 ± 0.02 ^c^	4.41 ± 0.04 ^a^
FLQ	6.43 ± 0.31 ^b^	6.32 ± 0.12 ^b^	12.67 ± 2.72 ^a^	17.94 ± 2.40 ^a^	11.47 ± 0.58 ^b^	20.75 ± 1.16 ^a^	35.60 ± 0.85 ^a^	11.87 ± 0.29 ^b^	35.42 ± 1.07 ^a^
AI	1.30 ± 0.06 ^a^	0.22 ± 0.02 ^b^	0.32 ± 0.01 ^b^	0.31 ± 0.06 ^a^	0.23 ± 0.00 ^b^	0.22 ± 0.01 ^b^	0.18 ± 0.01 ^a^	0.12 ± 0.01 ^b^	0.18 ± 0 ^a^
TI	1.94 ± 0.09 ^a^	0.45 ± 0.02 ^b^	0.49 ± 0.01 ^b^	1.07 ± 0.40 ^a^	0.64 ± 0.01 ^b^	0.56 ± 0.04 ^b^	1.15 ± 0.03 ^a^	0.16 ± 0.01 ^b^	1.13 ± 0.04 ^a^
HH	1.15 ± 0.06 ^c^	3.55 ± 0.24 ^a^	2.16 ± 0.16 ^b^	2.26 ± 0.05 ^a^	2.26 ± 0.11 ^a^	1.88 ± 0.04 ^b^	3.70 ± 0.17 ^b^	12.83 ± 0.31 ^a^	3.75 ± 0.22 ^b^
HPI	2.46 ± 0.11 ^c^	4.61 ± 0.33 ^a^	3.08 ± 0.14 ^b^	3.33 ± 0.61 ^b^	4.32 ± 0.07 ^a^	4.54 ± 0.30 ^a^	5.43 ± 0.20 ^b^	8.53 ± 0.39 ^a^	5.47 ± 0.12 ^b^
NVI	1.53 ± 0.08 ^b^	3.16 ± 0.29 ^a^	3.12 ± 0.14 ^a^	1.36 ± 0.83 ^a^	2.16 ± 0.05 ^a^	1.39 ± 0.35 ^a^	2.31 ± 0.10 ^b^	10.81 ± 0.84 ^a^	2.39 ± 0.06 ^b^

Values in the same row that do not share the same lowercase are significantly different (*p* < 0.05).

## Data Availability

The original contributions presented in this study are included in the article; further inquiries can be directed to the corresponding author.

## Supplementary Materials

The following supporting information can be downloaded at https://www.mdpi.com/article/10.3390/foods14142434/s1, Table S1: Fatty acid composition in the hepatopancreas, gonads, and muscle for three different habitats for female *Eriocheir sinensis* in the lower reach of the Yangtze River (mg/g total lipids); Figure S1: A typical representative chromatogram obtained during fatty acid analysis.
