# Supplementary material for: Nutritional Value of Female Eriocheir sinensis from Three Different Habitats in the Lower Reach of the Yangtze River with a Special Emphasis on Lipid Quality
Source: Foods. 2025 Jul 10;14(14):2434. doi: 10.3390/foods14142434 (PMC12294614; doi:10.3390/foods14142434)
Supplement: Supplementary file 1 [file foods-14-02434-s001.zip › foods-3708075-supplementary.pdf]

Supplementary Materials

Table S1. Fatty acid composition in the hepatopancreas, gonads and muscle for three different habitats female *Eriocheir sinensis* in the lower reach of the Yangtze River (mg/g total lipids)

Fatty acid	Hepatopancreas			Gonads			Muscles		
	Lake	Estuary	Pond	Lake	Estuary	Pond	Lake	Estuary	Pond
C12:0	0.63±0.02b	0.53±0.02c	0.81±0.04a	0.25±0.02b	0.21±0.01b	0.44±0.01a	0.47±0.04b	2.00±0.15a	0.09±0.00c
C13:0	0.37±0.01b	N.D.	0.49±0.07a	N.D.	N.D.	0.17±0.03a	N.D.	1.04±0.07a	0.07±0.00b
C14:0	12.77±0.53b	9.89±0.82b	27.30±3.12a	6.41±0.43b	5.77±0.41b	9.51±0.54a	1.52±0.10b	11.93±0.76a	2.26±0.12b
C15:0	0.04±0.00ab	0.03±0.00bc	0.04±0.01a	0.01±0.00b	0.03±0.00a	0.03±0.00a	0.01±0.00b	0.12±0.00a	0.01±0.00b
C16:0	157.42±1.74a	44.65±2.86c	54.03±4.15b	82.23±0.59b	78.12±4.07b	130.71±5.86a	84.66±5.02a	10.55±0.66b	84.48±2.68a
C17:0	3.12±0.31a	2.00±0.15b	2.38±0.18b	5.86±0.25a	2.00±0.07c	3.07±0.33b	3.98±0.11b	41.98±1.14a	4.53±0.17b
C18:0	24.66±1.53a	19.36±0.39b	19.09±0.76b	19.61±0.73b	24.41±0.58a	24.05±1.69a	51.36±1.25a	5.08±0.69b	53.73±1.67a
C20:0	1.94±0.06b	6.73±0.25a	1.73±0.68b	1.94±0.02a	0.79±0.03b	1.14±0.03b	0.88±0.01b	6.38±0.46a	0.83±0.05b
C21:0	0.65±0.02c	2.63±0.15a	1.52±0.12b	1.31±0.14c	2.09±0.12b	3.52±0.13a	0.96±0.06b	23.65±1.95a	1.64±0.07b
C22:0	10.77±0.64a	12.17±0.80a	11.50±1.16a	5.49±0.25c	7.09±0.12b	10.62±0.78a	11.81±0.37a	4.74±0.13b	12.09±0.11a
C23:0	10.21±0.53b	1.074±0.17b	14.86±0.90a	17.42±0.94b	21.25±1.23a	22.88±2.43a	31.01±1.46c	21.27±0.73b	34.03±1.11a
C24:0	1.36±0.10a	0.43±0.07b	1.68±0.26a	N.D.	4.13±0.43a	0.17±0.03b	N.D.	0.04±0.00b	0.18±0.03a
**ΣSFA**	228.20±1.68a	112.82±2.56c	140.32±4.43b	335.05±2.80a	148.96±4.36c	209.64±5.27b	187.89±7.13a	141.40±1.52b	195.32±2.70a
C14:1n-5	2.69±0.37a	2.18±0.12a	2.75±0.31a	0.65±0.12c	2.77±0.26a	1.11±0.16b	N.D.	6.65±0.71a	0.06±0.00b
C15:1n-5	N.D.	22.36±1.94a	4.78±0.61b	0.27±0.03c	1.27±0.07a	0.39±0.07b	0.27±0.02b	0.23±0.06b	0.93±0.07a
C16:1n-7	72.80±2.50b	65.94±3.92b	152.87±6.00a	56.17±3.71c	63.62±2.40b	83.13±2.38a	20.33±0.49b	26.44±0.27a	25.94±1.14a
C17:1n-7	4.19±0.37b	2.15±0.18c	5.04±0.41a	4.61±0.36a	3.15±0.44b	4.70±0.41a	2.88±0.23b	30.03±1.73a	3.53±0.37b
C18:1n-9c	0.78±0.01b	32.02±0.53a	1.35±0.08b	2.79±0.09b	3.32±0.11a	2.57±0.29b	1.34±0.08b	53.62±3.87a	1.35±0.13b
C18:1n-9t	215.54±11.39a	89.07±4.43c	148.56±19.87b	89.39±6.67a	141.35±10.78a	154.04±3.77a	142.93±2.08a	54.92±4.42b	147.12±13.20a
C20:1n-9	13.66±1.41a	9.85±0.52b	13.53±0.98a	3.66±0.21c	30.68±1.08a	6.49±0.61b	4.61±0.21b	12.79±0.81a	5.02±0.80b
C22:1n-9	8.20±0.69a	1.85±0.10b	2.92±0.04b	1.55±0.07b	3.12±0.12a	1.18±0.13c	1.18±0.03b	19.46±0.67a	1.00±0.04b
C24:1n-9	1.81±0.17a	1.05±0.02c	1.36±0.05b	3.13±0.11b	2.49±0.12c	3.71±0.24a	3.06±0.11b	48.94±0.96a	3.54±0.31b
**ΣMUFA**	319.70±15.83a	226.50±2.57b	328.89±2.49a	162.25±6.98c	251.80±9.78b	505.52±29.63a	176.64±2.71b	253.11±2.44a	188.54±12.49b
C18:2n-6t	N.D.	1.35±0.14a	0.80±0.08b	N.D.	1.08±0.07a	0.68±0.10b	N.D.	6.42±0.71a	0.30±0.02b
C18:2n-6c	88.16±4.701a	95.43±2.87a	66.28±6.24b	53.75±2.80b	73.79±4.84a	76.74±5.12a	59.97±1.66a	35.85±1.76c	56.10±2.14b
C18:3n-6	0.41±0.02b	0.19±0.01c	1.57±0.07a	1.01±0.12b	8.52±0.46a	1.32±0.08b	0.49±0.05a	0.15±0.01b	0.50±0.01a
C18:3n-3	12.70±0.97b	25.40±1.58a	13.19±0.83b	14.24±0.22c	29.22±0.59a	23.39±1.93b	8.86±0.44b	48.16±0.65a	9.53±0.77b
C20:2n-6	2.34±0.27a	0.93±0.01b	2.67±0.64a	0.75±0.12a	0.14±0.01c	0.42±0.07b	0.83±0.01b	3.63±0.42a	0.97±0.08b

Continued Table S1.

C20:3n-6	1.97±0.05b	1.55±0.19b	2.86±0.18a	0.78±0.04b	1.74±0.31a	1.32±0.28a	0.61±0.02b	19.49±0.82a	0.73±0.04b
C20:3n-3	3.01±0.11b	4.48±0.08a	3.11±0.18b	2.50±0.08c	3.94±0.07a	4.21±0.22a	3.61±0.30b	44.21±1.17a	3.69±0.18b
C20:4n-6	N.D.	N.D.	0.51±0.06a	N.D.	0.07±0.00b	0.10±00a	N.D.	4.36±0.20a	N.D.
C22:2n-6	3.51±0.20a	2.08±0.68b	3.28±0.58a	1.38±0.38b	0.46±0.16c	2.44±0.27a	0.95±0.14b	20.39±0.95a	1.15±0.19b
C20:5n-3	21.03±1.26b	12.40±2.87c	25.30±1.89a	45.47±2.86b	35.35±2.22c	75.44±1.31a	115.60±2.69b	30.47±2.13c	121.48±3.58a
C22:6n-3	62.00±5.48a	19.37±5.33c	56.79±20.73a	78.25±3.72a	32.07±2.93b	75.66±3.02a	127.60±8.76a	47.28±1.19b	129.06±3.27a
**∑PUFA**	195.17±10.68a	163.22±3.34b	176.39±16.60b	198.18±5.34b	186.29±6.08b	261.76±9.78a	318.56±12.41a	260.47±4.85b	323.55±10.01a
**∑UPA**	514.88±26.09a	389.72±5.10b	505.29±33.11a	360.44±34.74b	438.09±9.35b	767.29±19.85a	513.59±4.94a	495.20±14.49a	512.10±6.48a
**∑n-3PUFA**	98.75±6.25a	61.66±2.18b	98.40±2.159a	140.48±5.06b	100.59±4.89c	178.71±6.14a	255.69±10.77a	170.14±3.77b	263.77±7.79a
**∑n-6PUFA**	96.41±4.75a	101.55±3.03a	77.99±5.09b	57.70±2.27b	85.69±5.26a	83.05±4.83a	62.87±2.74b	90.33±1.17a	59.78±2.24b
**∑LC-PUFA**	93.89±5.88a	40.83±2.85b	94.54±9.37a	12916.64±504.75b	73.65±4.53c	159.61±4.36a	249.23±10.89a	169.87±4.37b	257.12±7.15a
**EPA+DHA**	83.04±5.77a	31.77±0.822b	82.09±10.58a	123.72±4.99b	67.42±4.91c	151.10±4.08a	243.21±11.03a	77.75±2.16b	250.54±6.85a

Note: ND: not detected; Values in a same row that do not share a same superscript are significantly different (p<0.05). Abbreviation: SFA, saturated fatty acid; MUFA, monounsaturated fatty acid; PUFA, polyunsaturated fatty acid; UPA, unsaturated fatty acid.

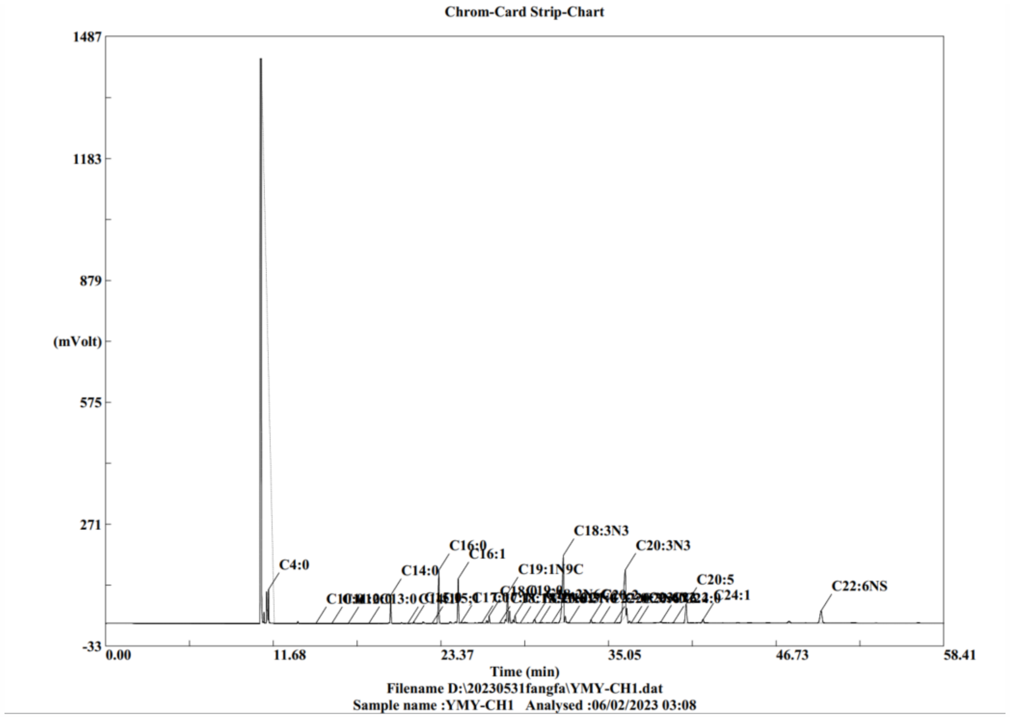

Figure S1. A typical representative chromatogram obtained during fatty acid analysis.
